# Protective Effect of *Punica granatum* L. against Serum/Glucose Deprivation-Induced PC12 Cells Injury

**DOI:** 10.1155/2013/716730

**Published:** 2013-07-07

**Authors:** Fatemeh Forouzanfar, Amir Afkhami Goli, Elham Asadpour, Ahmad Ghorbani, Hamid Reza Sadeghnia

**Affiliations:** ^1^Pharmacological Research Center of Medicinal Plants, Department of Pharmacology, School of Medicine, Mashhad University of Medical Sciences, Mashhad 917794-8564, Iran; ^2^School of Veterinary Medicine, Ferdowsi University of Mashhad, Mashhad, Iran; ^3^Neurocognitive Research Center, Department of Modern Sciences and Technology, School of Medicine, Mashhad University of Medical Sciences, Mashhad 917794-8564, Iran

## Abstract

The discovery and development of natural products with potent antioxidant, anti-inflammatory, and antiapoptotic properties have been one of the most interesting and promising approaches in the search for the treatment of many neurodegenerative diseases including ischemic stroke. Serum/glucose deprivation (SGD) has served as an excellent in vitro model for the understanding of the molecular mechanisms of neuronal damage during ischemia and for the development of neuroprotective drugs against ischemia-induced brain injury. Recent studies suggested that pomegranate (*Punica granatum* L.) or its active constituents exert pharmacological actions such as antioxidant, anti-inflammatory, and neuroprotective properties. Therefore, in this study we investigated the possible protective effects of different extracts of pomegranate against SGD-induced PC12 cells injury. Initially, the cells were pretreated with different concentrations of pulp hydroalcoholic extract (PHE), pulp aqueous extract (PAE) and pomegranate juice (PJ) for 2 h and then deprived of serum/glucose (SGD) for 6 and 12 h. SGD caused a significant reduction in cell viability (measured by the MTT assay) after 6 and 12 h, as compared with control cells (*P* < 0.001). Pretreatment with PHE, PAE, and PJ significantly and concentration-dependently increased cell viability following SGD insult for 6 and 12 h. A significant increase in DNA damage (measured by the comet assay) was seen in nuclei of cells following SGD for 12 h (*P* < 0.001). In control groups, no significant difference was seen in DNA damage between PHE, PAE, and PJ-pretreated and vehicle-pretreated PC12 cells (*P* > 0.05). PHE, PAE, and PJ pretreatment resulted in a significant decrease in DNA damage following ischemic insult (*P* < 0.001). This suppression of DNA damage by PHE, PAE and PJ was found to be concentration dependent. These data indicate that there is a cytoprotective property in PHE, PAE, and PJ under SGD condition in PC12 cells, suggesting that pomegranate has the potential to be used as a new therapeutic strategy for neurodegenerative disorders.

## 1. Introduction

In spite of remarkable promotion in the prevention and treatment of cerebral ischemia, stroke still remains one of the most important causes of death and cripple in the aged population [1]. Designing the neuroprotective drugs and studying the cytoprotective effects of valid component could be done with an efficient in vitro model like serum/glucose deprivation (SGD) neuronal damage which could characterize the molecular mechanism of brain injury during cerebral ischemia [2, 3].

*Punica granatum *L., (family Punicaceae), is a fruit-bearing deciduous shrub or small tree growing between five and eight meters tall. The pomegranate is native to the region of Persia and the western Himalayan range [4] and has been cultivated in Iran, Mediterranean region, California, and many other regions [5]. In Ayurvedic medicine the pomegranate is considered “a pharmacy unto itself” and is used as an antidiabetic, antiparasitic agent and to heal gastrointestinal disorders such as aphthae, diarrhea, and ulcers [6]. Recently, it has been shown that pomegranate possesses several pharmacological activities such as antioxidant [6–8], anticarcinogenic [7, 8], and anti-inflammatory properties [9]. It is documented that consumption of pomegranate appears to correlate with treatment and preventing widespread range of diseases such as cancer [7, 8], diabetes [10], cardiovascular disease [11, 12], rheumatoid arthritis, and ulcerative colitis [9]. The neuroprotective effects of pomegranate have been shown in hypoxic-ischemic brain injuries [13, 14], A*β*-induced oxidative stress and learning and memory deficits, and also H_2_O_2_-induced oxidative stress in PC12 cells [15].

Since it has been shown that *Punica granatum *has many beneficial consequences correlating with its antioxidant effect, we decided to study its protective effect against the SGD-induced PC12 cells injury.

## 2. Material and Methods

### 2.1. Cell Line and Reagents

A PC12 cell line was obtained from Pasteur Institute (Tehran, Iran). High glucose Dulbecco's modified Eagles medium (DMEM, 4.5 g/L), glucose-free DMEM (0 g/L), and fetal calf serum (FCS) were purchased from Gibco (Carlsbad, CA). The Folin-Ciocalteu reagent, the 3-(4,5-dimethylthiazol-2-yl)-2,5-diphenyl tetrazolium (MTT), and other cell culture materials were purchased from Sigma (St. Louis, MO). Low melting point (LMP) agarose and normal melting point (NMP) agarose were obtained from Fermentas (Glen Burnie, MD). Other chemicals mainly ethylene diamine tetraacetic acid disodium salt (Na_2_EDTA), Tris (hydroxymethyl) aminomethane (Trizma base), t-octylphenoxy polyethoxyethanol (Triton X-100), dimethyl sulfoxide (DMSO), sodium lauroyl sarcosinate (sarkosyl, SLS), and ethidium bromide were purchased from Merck (Darmstadt, Germany).

### 2.2. Cell Culture

PC12 cells were cultured in high glucose DMEM (4.5 g/L) supplemented with 10% FCS and 100 units/mL of penicillin/streptomycin. All cells were maintained in a humidified atmosphere (90%) containing 5% CO_2_ at 37°C.

### 2.3. Induction of Cell Death by Serum/Glucose Deprivation

For SGD-induced cytotoxicity, PC12 cells were seeded overnight and then were subjected to SGD for 6 and 12 h by replacing the standard culture medium (high glucose DMEM, 4.5 g/L) with the glucose-free DMEM (0 g/L), supplemented with 100 U/mL penicillin and 100 U/mL streptomycin [16].

### 2.4. Preparation of Pulp Hydroalcoholic Extract (PHE), Pulp Aqueous Extract (PAE) and Pomegranate Juice (PJ)

Pomegranate fruits were collected from Saveh region (center of Iran) and authenticated by herbarium of Ferdowsi University of Mashhad (Mashhad, Iran; voucher specimen no. 311-0203-1).

The pulps and peels of pomegranate were washed, dried, and crushed to a powder with an electric microniser. The powdered pulps were then shaken with ethanol (70%) and water for 2 days. The resulting extracts were then filtered and concentrated under reduced pressure to get pulp hydroalcoholic extract (PHE) and pulp aqueous extract (PAE), respectively. The yield was found to be about 36% w/w. To obtain pomegranate juice extract (PJ), the seeds were separated and ground to obtain juice. The juice was then filtered and dried under reduced pressure (yielding 4.1% w/w). The extracts were kept at −20°C until use.

Stock solutions of PHE and PAE were prepared in dimethyl sulfoxide (DMSO), and desired concentrations were made from the stock using complete medium. Stock and working solutions of PJ were prepared in complete medium.

### 2.5. Total Phenolics Measurement

The total phenolics concentration of PHE, PAE, and PJ was determined by the Folin-Ciocalteu method as previously described [17]. Briefly, 50 mg of the dried extract was extracted with 100 mL of acidified methanol : water (60 : 40 v/v, 0.3% HCl) before subsequent filtration. 100 *μ*L of filtrate was then mixed with equal amounts of the Folin-Ciocalteu reagent, while 2.0 mL of sodium bicarbonate was added and mixed thoroughly. Absorbance was measured at 750 nm, and the values were derived from a standard curve prepared using tannic acid (0–1.0 mg/mL in acidified methanol : water) after 2 hours. Values were expressed as tannic acid equivalents (TAE, mg/g dry mass).

### 2.6. Cell Proliferation (MTT) Assay

MTT was used to identify viable cells which reduce it to a violet formazan [18]. PC12 cells (5000/well) were seeded out in 96-well tissue culture plates, and after 24 h, the cells were pretreated with PHE, PAE and PJ (6.25–800 *μ*g/mL) for 2 h and then incubated simultaneously for another 6 or 12 h in serum and glucose free (SGD) condition. These doses were chosen based on IC_50_ (concentration of 50% inhibition) calculated from earlier experiments. Blank and solvent controls were treated identically. At 6 and 12 h after SGD insult, MTT solution in phosphate-buffered saline (PBS, 5 mg/mL) was added to a final concentration of 0.05%. After 2 h, the formazan precipitate was dissolved in DMSO containing 10% glycine buffer (pH = 10.5). The microplates were then gently shaken in the dark for 30 min, and the absorbance at 570 and 620 nm (background) was measured using a StatFAX303 plate reader.

All experiments were carried out in triplicate; the percentage of viable cells was calculated as the mean ± SEM with controls set to 100%. Morphological deformations of the cells were also examined.

### 2.7. Single Cell Gel Electrophoresis (SCGE, Comet) Assay

The alkaline SCGE assay was conducted based on the method described previously [19]. Briefly, PC12 cells (3 × 10^5^) were incubated for 2 h with three different concentrations of PHE, PAE, and PJ (6.25, 400, and 800 *μ*g/mL) and subjected to SGD for 12 h in which the same treatments were applied. After removing the medium, the cells were washed three times with cold PBS, harvested and centrifuged at 3000 rpm for 5 min at 4°C. The pellets were then resuspended in PBS at a cell density of 1 × 10^5^. For the comet assay, 100 *μ*L NMP agarose was quickly layered on conventional slides, covered with a cover slip, and then the slides were placed on ice to allow agarose to gel. 10 *μ*L of the cell suspension, prepared as above, was mixed with 100 *μ*L LMP agarose, and the mixture was quickly layered over the NMP agarose layer after removal of the cover slip. Finally, another layer of LMP agarose was added on top. The slides were immersed immediately in a chilled lysing solution (pH = 10) made up of 2.5 M NaCl, 100 mM Na_2_EDTA, 10 mM Trizma, 1% sarkosyl, 10% DMSO, and 1% triton X-100 and kept at 0°C in the dark overnight. Then, the slides were placed on a horizontal gel electrophoresis platform and covered with a prechilled alkaline solution made up of 300 mM NaOH and 1 mM Na_2_EDTA (pH > 13). They were left in the solution in the dark at 0°C for 40 min and then electrophoresed at 0°C in the dark for 30 min at 25 V and approximately 300 mA. The slides were rinsed gently three times with 400 mM Trizma solution (adjusted to pH 7.5 by HCl) to neutralize the excess alkali, stained with 50 *μ*L of 20 *μ*g/mL ethidium bromide and covered with a cover slip. For comet analysis, 150 nuclei were randomly selected from three replicated slides (50 nuclei on one slide), examined and photographed through a fluorescence microscope (Nikon, Japan), at 400x magnification equipped with an excitation filter of 520–550 nm and a barrier filter of 580 nm. Undamaged cells resemble an intact nucleus without a tail, and damaged cells have the appearance of a comet. The percent of DNA in the comet tail (% tail DNA), which is an estimation of DNA damage, was analyzed using the computerized image analysis software (CASP software). The experiments were done as triplicate.

### 2.8. Statistical Analysis

The results are presented as the mean ± standard error (SEM). The values were compared using the one-way analysis of variance (ANOVA) followed by Tukey's post hoc test for multiple comparisons. The *P* values less than 0.05 were considered to be statistically significant.

## 3. Results

### 3.1. Total Phenolics Content of PHE, PAE and PJ

Polyphenols including hydrolysable tannins and ellagitannins account for the main known antioxidant properties of pomegranate [20]. The PHE, PAE, and PJ were found to contain 353 ± 8, 224 ± 5, and 119 ± 6 mg/L total polyphenolics expressed as tannic acid equivalents (TAE, mg/g of TAE), respectively.

### 3.2. PHE, PAE, and PJ Dose-Dependently Inhibit SGD-Induced Cell Death

**Figure 1:**
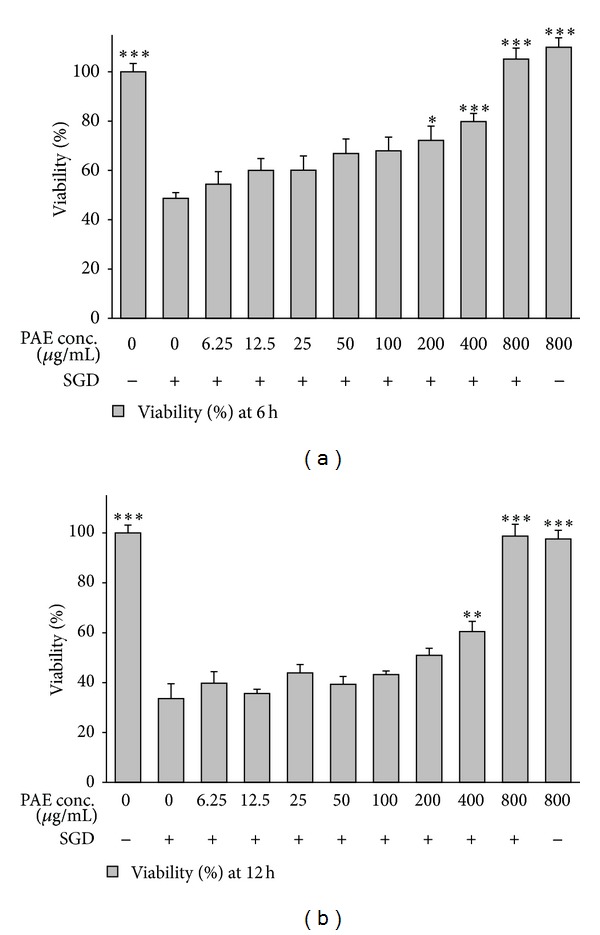
Effects of pulp aqueous extract (PAE) on PC12 cells viability exposed to SGD (serum/glucose deprivation) for 6 and 12 h. The percentage cell viability (quantitated by MTT assay) was normalized against the control (0 *μ*M). Mean and SEM of the three independent experiments were shown. *P < 0.05, **P < 0.01, ***P < 0.001 as compared with control.

**Figure 2:**
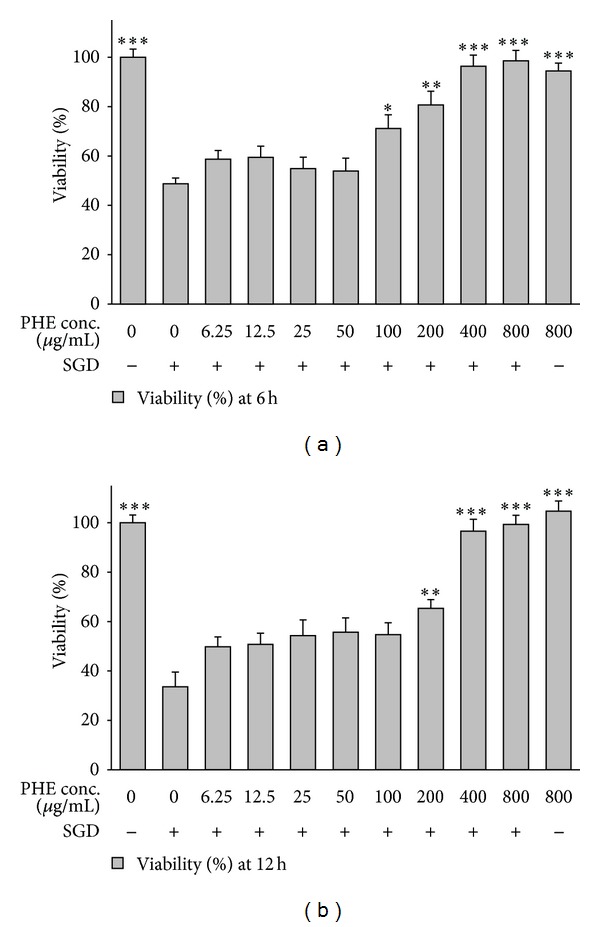
Effects of pulp hydroalcoholic extract (PHE) on PC12 cells viability exposed to SGD (serum/glucose deprivation) for 6 and 12 h. The percentage cell viability (quantitated by MTT assay) was normalized against the control (0 *μ*M). Mean and SEM of the three independent experiments were shown. *P < 0.05, **P < 0.01, ***P < 0.001 as compared with control.

**Figure 3:**
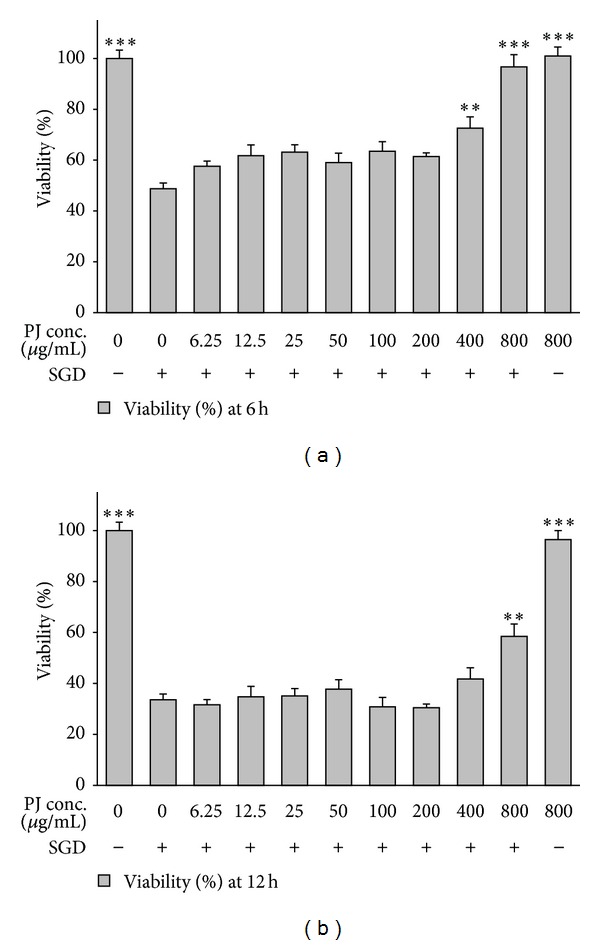
Effects of PJ (pomegranate juice) on PC12 cells viability exposed to SGD (serum/glucose deprivation) for 6 and 12 h. The percentage cell viability (quantitated by MTT assay) was normalized against the control (0 *μ*M). Mean and SEM of the three independent experiments were shown. **P < 0.01, ***P < 0.001 as compared with control.

To examine the probable toxic effects of pomegranate extracts, PC12 cells were incubated with high concentrations of PHE, PAE, and PJ (800 *μ*g/mL) alone, and the viability was determined 6 and 12 h after treatment. No significant toxic effect on cell viability was seen following treatment with these extracts for 6 and 12 h (Figures 1–3).

SGD caused a significant reduction in cell viability after 6 and 12 h, as compared with control group (Figures 1–3). As shown in Figures 2(a)-2(b), treatment with PHE resulted in significant and concentration-dependent increase in cell viability following ischemic insult for 6 h (*P* < 0.05 at 100 *μ*g/mL) and 12 h (*P* < 0.01 at 200 *μ*g/mL). 

A significant and concentration-dependent increase in cell viability was seen following treatment with PAE after ischemic insult for 6 h (*P* < 0.05 at 200 *μ*g/mL) and 12 h (*P* < 0.01 at 400 *μ*g/mL) (Figures 1(a)-1(b)). Pretreatment with PJ also significantly and dose-dependently decreased SGD-induced cell death following 6 and 12 h (*P* < 0.01 at 400 *μ*g/mL and *P* < 0.01 at 800 *μ*g/mL, resp., Figures 3(a)-3(b)).

### 3.3. PHE, PAE, and PJ Significantly Decrease SGD-Induced DNA Damage

**Figure 4:**
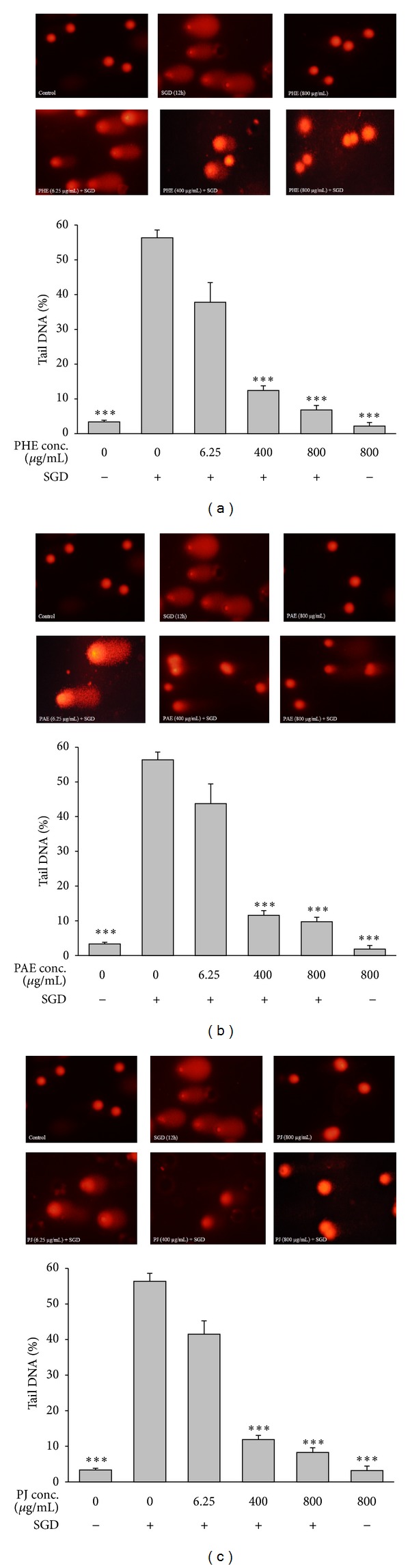
Representative micrographs of comets from PC12 cells of different treatment groups (top panel); %tail DNA (as an indicator of DNA damage) induced by serum/glucose deprivation (SGD) in PC12 cells after 12 h (bottom panel). Cells were pretreated with different concentrations of pulp hydroalcoholic extract (PHE), pulp aqueous extract (PAE), and pomegranate juice (PJ). All data were represented as the means ± SEM of three independent experiments. ***P < 0.001 as compared with SGD.

In this study, %tail DNA was measured as an indicator of DNA damage. The results showed that exposure of PC12 cells to SGD significantly increased DNA fragmentation (DF), compared with control cells (*P* < 0.001, Figure 4). A significant decrease in SGD-induced DF was seen following pretreatment with high doses (400, 800 *μ*g/mL) of PHE, PAE, and PJ (*P* < 0.001, Figure 4).

## 4. Discussion

Ischemic stroke is a third leading cause of death and a major cause of disability in industrialized countries. Currently, therapeutic options for treatment of stroke are limited, and much more attempts are being made to identify novel neuroprotective agents with antiapoptotic properties [23, 24].

Oxidative damage and induction of apoptotic cell death is a characteristic feature of many neurodegenerative diseases including ischemic stroke [25]. In this study, the protective effect of *Punica granatum* (pomegranate) against serum/glucose deprivation- (SGD-) induced cell death in PC12 cells is investigated here for the first time. We showed that pomegranate exhibits no cytotoxicity on PC12 cells at used concentrations (up to 800 *μ*g/mL). Moreover, we found that pretreatment with different pomegranate extracts significantly decreased cell loss and DNA damage under SGD condition in a concentration-dependent manner, which showed that neuroprotective activity of pomegranate probably mediated through attenuation of DNA damage.

SGD is regarded as a reliable model mimicking the effects of ischemia in vitro. Lorenz et al. compared the validity of SGD in vitro, with permanent middle cerebral artery occlusion (MCAO) in vivo. SGD for 4 h induced half-maximal neuronal cell death, while MCAO for the same period resulted in significant neuronal loss, suggesting the validity of SGD as a suitable stroke-like in vitro model [26]. In our study, about 50% and 65% cell losses were seen under SGD condition for 6 h and 12 h, respectively, which are in agreement with previous reports [16, 26, 27]. The previous studies also correspond to our findings that SGD for 18 or 24 h caused significant DNA fragmentation in the nuclei of PC12 cells [28, 29] suggesting apoptotic/necrotic cell death.

It is generally believed that pathophysiological basis of many neurodegenerative disorders such as ischemic stroke is probably mediated through glutamate excitotoxicity, overproduction of oxygen free radicals (ROS), and resulting oxidative damage to cellular macromolecules, including membrane lipids, proteins and nucleic acids, neuroinflammation, and induction of delayed cell death or apoptosis [30].

*P. granatum* has potent antioxidant and anti-inflammatory properties. According to previous studies, pomegranate is an important source of polyphenols including phenolic acids, ellagic tannins (punicalin, punicalagin, gallagic, and ellagic acid), flavnoids (anthocyanins, catechins, rutin, epigallocatechin-3-gallate), and anthocyanins (delphinidin, cyaniding, and pelargonidin) [31–33], which possess potent antioxidant and radical scavenger properties [21, 34, 35]. Recent studies have shown that neuroprotection afforded by pomegranate could be attributed to the antioxidant or other properties of these phenolic compounds. Therefore, the total phenolic concentration of PHE, PAE, and PJ was determined by the Folin-Ciocalteu method. The results indicated that PHE displayed the highest amount of polyphenol compounds (353 mg/g as TAE) rather than PAE (224 mg/g) or PJ (119 mg/L). These results in agreement with previous reports showed that pomegranate peels and pulps have higher total phenolics and antioxidant activity than juice [36, 37]. Yasoubi at al. also identified that the concentration of phenolics in the pomegranate peel extracts depends on the polarity of solvent. It was shown that more phenolic materials are present in the alcoholic extract than in aqueous extract [38]. It has been shown that hydrolyzable tannins (HT) account for about 92% of pomegranate antioxidant activity [39]. The predominant pomegranate HT is punicalagin, which is responsible for about half of pomegranate total antioxidant capacity [40, 41] but its water solubility has been reported to be very low (about 0.2–1.0%) [42]. Both flavonoids and tannins are also most abundant in pomegranate pulp extracts (i.e. PHE and PAE). Our study demonstrated that PHE has the most potent protective effect against SGD than PAE and PJ, which could be as a result of the higher phenolic contents with less water solubility such as ellagic tannins like gallic acid and punicalagin [43]. On the other hand, the protective effects of PJ against SGD could be explained as the consequence of anthocyanins, which are the water-soluble pigments responsible for the bright red color of pomegranate [32, 44].

As mentioned, pomegranate has appreciable antioxidant and free radical scavenger properties. Therefore, the protective activity of pomegranate against SGD seen in this study probably mediated through attenuation of oxidative damage. These findings are in agreement with other reports on PC12 cell line, showing that the ethanol extract of *P. granatum* mitigated H_2_O_2_-induced oxidative stress in PC12 cells. In addition, the extract inhibited learning and memory deficits and neuronal cell death caused by A*β*-induced oxidative stress in mouse hippocampus, and it also improves behavior and decreases hippocampal amyloid load in a mouse model of Alzheimer's disease [15, 45]. Polyphenol-rich pomegranate juice also protected the neonatal mouse brain against hypoxic-ischemic injury via caspase-3 inhibition [14]. It has been shown that pomegranate flowers (PGFs) supplementation decreases oxidative stress and ameliorates impairment in learning and memory performances in diabetic rats [46].

Punicalagin extracted from PJ and pomegranate peel inhibits LDL oxidation and atherosclerosis development in mice [47]. Daily supplementation with pomegranate in diet could also control sign and symptoms in patients with rheumatoid arthritis due to its antioxidant potency [48]. It is shown that pomegranate constituents afford chemoprevention against hepatocarcinogenesis through antioxidant properties [49].

The flavonoid rich fractions of pomegranate fruit extract have also been shown to exert antiperoxidative effect by decreasing the concentrations of malondialdehyde and hydroperoxides and enhancing the activities of enzymes catalase, superoxide dismutase, glutathione peroxidase, and glutathione reductase in the liver [50, 51]. It is concluded that the antioxidative characteristics of pomegranate unique phenolic compounds, punicalagin and gallic acid, could be related, at least in part, to their stimulatory effect on macrophage paraoxonase 2 (PON 2) expression, a phenomenon which was shown to be associated with activation of the transcription factors PAPR-*γ* and AP-1 [22]. The other mechanisms that could be attributed to pomegranate protective effects are mast cell stabilizing and anti-inflammatory actions. The *Punica granatum* potency in ameliorating colitis is attributed to its ellagic acid rich fraction [21]. Other studies have shown that prodelphinidins which are present in pomegranate fruit inhibit cyclooxygenase-2 (COX-2) and lipoxygenase activity and production of prostaglandins E_2_ (PGE_2_) in vitro suggesting the anti-inflammatory properties of pomegranate [52]. Recently it has been shown that pomegranate extract inhibited the expression of inflammatory cytokines IL-1*β* and IL-6 in adjunctive periodontal therapy [53]. NF-*κ*B is an important transcriptional regulator of inflammatory cytokines gene expression and plays a crucial role in immune and inflammatory responses. It has been shown that pretreatment with pomegranate extract inhibited the degradation of I*κ*B*α* and nuclear translocation of NF-*κ*B in KU812 cells [54]. Antioxidant potency of a methanolic pomegranate fruit peel extract (PPE) and its relation with antiproliferative and apoptotic effects on MCF-7 human breast cancer cells have been evaluated, and the results showed that it has significant antioxidant and apoptotic effects [55]. The hydroxybenzoic acid constituents of pomegranate (gallic and ellagic acids) induced p53/p21 expression, G1 arrest and apoptosis in bladder cancer cells [56], as well as human DU-145 prostate cancer cell line [57]. In addition, it has been shown that pomegranate juice metabolites, ellagic acid and urolithin A, synergistically inhibit cell proliferation and induce cell cycle arrest and apoptosis in DU-145 and PC-3 androgen-independent prostate cancer cells [58]. It is also reported that other active constituents of pomegranate like proanthocyanidins and anthocyanidins have anticarcinogenic effects through antiangiogenic, antimutagenic activities [59, 60] and inhibition of cyclooxygenase activity, nitric oxide production, and epidermal growth factor receptors [61]. A recent study also provides evidence that pomegranate phytochemicals exert chemoprevention against diethylnitrosamine-induced rat hepatocellular carcinoma through antiproliferative and proapoptotic mechanisms by modulating NF-*κ*B and Wnt/*β*-catenin signaling pathways [62]. 

**Figure 5:**
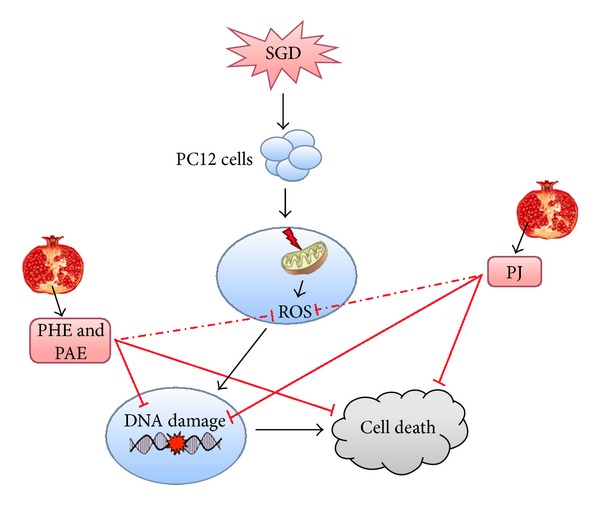
Schematic representation of the protective effects of pomegranate extracts against serum/glucose deprivation- (SGD-) induced PC12 cells injury. SGD may induce mitochondrial dysfunction, calcium overload, overproduction of reactive oxygen species (ROS), and resulting oxidative damage to cellular macromolecules, including membrane lipids, proteins and DNA, neuroinflammation, and induction of cell death. Antioxidant (by scavenging of free radicals or upregulation of paraoxonase 2 expression, leading to inhibition of oxidative damage) [21, 22], anti-inflammatory, neuroprotective, and antiapoptotic properties (by inhibition of cyclooxygenase 2 expression, leading to decreased production of prostaglandins and by decreased transcription of proinflammatory cytokines such as tumor necrosis factor *α* (TNF-*α*), interleukin 6 (IL-6), and IL-1*β* through the modulation of PPAR-*γ*, NF-*κ*B, AP-1, or MAPK signaling) [6, 9, 14] of pomegranate phytochemicals may block these pathways. PAE: aqueous extract of pomegranate peel and pulp, PHE: hydroalcoholic extract of pomegranate peel and pulp, PJ: pomegranate juice, ↓: activation, ⊥: inhibition.

At last, we conclude that *Punica granatum* has protective effects against SGD-induced cytotoxicity in PC12 cells through its antioxidant activity and subsequent DNA damage, suggesting its antiapoptogenic properties. But further studies are needed to elucidate the possible underlying mechanisms of these beneficial effects (Figure 5), as well as whether substances in pomegranate extract may be useful in stroke should be considered.
